# Supplementary material for: Soluble ST2 as a biomarker for predicting severe adverse events among pediatric patients with Mycoplasma pneumoniae pneumonia
Source: PLoS One. 2026 Apr 17;21(4):e0347651. doi: 10.1371/journal.pone.0347651 (PMC13089705; doi:10.1371/journal.pone.0347651)
Supplement: S1 File — (PDF) [file pone.0347651.s001.pdf]

Definitions and classifications of Severe Adverse Events and Complications

- **Severe adverse events** were defined as in-hospital death, ICU admission, diagnosis of sepsis, or use of extracorporeal membrane oxygenation.

1. In-hospital death is death that occurred during hospitalization.
2. In our hospital, a patient is typically admitted to the ICU if at least one of the following conditions is present: (1) $FiO_2 \geq 0.6$, $SaO_2 \leq 0.92$, (2) shock and/or impaired consciousness, (3) tachypnea and tachycardia with severe respiratory distress or signs of exhaustions, with or without elevated $PaCO_2$, (4) recurrent apnea, or slow and irregular breathing, and (5) other conditions requiring further monitoring and treatment (e.g. renal failure, severe thrombocytopenia)
3. The definition of sepsis follows the Third International Consensus Definitions for sepsis as life-threatening organ dysfunction caused by a dysregulated host response to infection, where organ dysfunction can be identified as ≥ 2 points on the Phoenix Sepsis Score in children with suspected infection [1].
4. The use of ECMO is mainly indicated for patients requiring respiratory support due to: (1) PaO_2/FiO_2 < 60-80 mmHg due to severe respiratory failure; (2) failure of conventional ventilation and/or other rescue therapies; (3) high ventilator settings (e.g., mean airway pressure > 20 - 25 cm H_2O during conventional ventilation or > 30 cm H_2O during high-frequency ventilation, or signs of iatrogenic barotrauma). ECMO is also indicated for circulatory support in cases such as cardiogenic shock unresponsive to standard medication, with low SBP < 50 mmHg, urine volume < 1 ml / (kg·h), lactic acidosis, central venous oxygen saturation < 0.6, altered mental status due to low cardiac output, and refractory septic shock with an epinephrine dose > 1 μg / (kg·min) or vasoactive-inotropic score >100.

Pulmonary and extrapulmonary complications were defined according to the Chinese guidelines for the management of community-acquired pneumonia in children (2024 revision) [2].

- **Pulmonary complications** included acute respiratory distress syndrome (ARDS), respiratory failure, pulmonary necrosis, pleural effusion, plastic bronchitis, pulmonary embolism, and pulmonary atelectasis.

1. Acute respiratory distress syndrome is diagnosed according to the PALICC-2 criteria [3]. Respiratory failure must develop within 7 days of a known clinical insult, which cannot be fully explained by cardiac failure or fluid overload. The presence of new parenchymal opacities on chest imaging is not due to atelectasis or effusion. Hypoxemia is stratified by the mode of respiratory support. For patients on invasive mechanical ventilation, hypoxemia is assessed using the Oxygenation Index ≥ 4 or Oxygen Saturation Index ≥ 5. For those on non-invasive ventilation (with a full-face interface and CPAP PEEP ≥ 5 cm H_2O) or high-flow nasal cannula, hypoxemia is diagnosed based on $PaO_2/FiO_2 \leq 300$ or $SpO_2/FiO_2 \leq 250$.
2. Respiratory failure is diagnosed: (1) underlying etiology—identification of the primary or contributory condition causing respiratory failure; (2) clinical manifestations—presence of increased work of breathing (e.g., tachypnea with or without cyanosis) or irregular breathing patterns; (3) arterial blood gas (ABG) criteria — for Type I (hypoxemic) respiratory failure, PaO_2 < 60 mmHg with normal or low $PaCO_2$; for Type II (hypercapnic) respiratory failure, PaO_2 < 60 mmHg and $PaCO_2$ > 50 mmHg. For patients on supplemental oxygen, a PaO_2/FiO_2 ratio < 200 mmHg suggests significant intrapulmonary shunting (>20%), while a ratio > 300 mmHg is generally considered normal.
3. Pulmonary necrosis is diagnosed via CT examination, showing multiple thin-walled cavities with low density, containing air or fluid, occurring in areas of pulmonary consolidation.
4. Pleural effusion is diagnosed via chest ultrasound, CT or X-ray examination. Imaging findings include blunting of the costophrenic angle on X-ray, anechoic or

septated fluid collections on ultrasound, and crescent-shaped opacities in the dependent pleural space on CT.

5. Plastic bronchitis is diagnosed based on the expectoration or bronchoscopic removal of bronchial tree-shaped casts.
6. Pulmonary embolism is diagnosed based on CT pulmonary angiography showing filling defects within the pulmonary arteries, reduction or absence of distal vascular branches, and wedge-shaped pulmonary lesions.
7. Pulmonary atelectasis: Diagnosed via CT scan, showing signs of volume loss, such as displacement of interlobar fissures toward the area of collapse, crowding of pulmonary vessels and bronchi within the affected region, and compensatory hyperinflation of the remaining lung tissue.

- **Extrapulmonary complications** were classified as follows:

1. Nervous system involvement includes encephalitis, acute disseminated encephalomyelitis, transverse myelitis, Guillain-Barré syndrome, and cerebral infarction, with encephalitis being the most common. These conditions should be considered when children with pneumonia present with symptoms such as convulsions or altered consciousness.
2. Circulatory system involvement includes intracardiac thrombosis, septic shock, myocarditis, pericarditis, Kawasaki disease, arterial embolism, and venous thrombosis. These conditions should be considered when children with pneumonia have abnormal cardiac biomarkers. Particular attention should be paid to these complications in children with severe or refractory MPP.
3. Hematological system involvement includes immune thrombocytopenia, autoimmune hemolytic anemia, hemophagocytic syndrome, and disseminated intravascular coagulation. These complications should be considered when there is a significant decrease in peripheral blood cell lines.
4. Skin and mucosal lesions include urticaria, Henoch-Schönlein purpura, erythema multiforme, Stevens-Johnson syndrome, toxic epidermal necrolysis, and Mycoplasma pneumoniae-induced rash and mucositis (MIRM).
5. Other manifestations include glomerulonephritis, acute kidney injury, liver failure,

acute pancreatitis, arthritis, and rhabdomyolysis.

[1] Schlapbach LJ, Watson RS, Sorce LR, Argent AC, Menon K, Hall MW, et al. International Consensus Criteria for Pediatric Sepsis and Septic Shock. JAMA. 2024;331(8):665-674. doi:10.1001/jama.2024.0179

[2] Subspecialty Group of Respiratory, the Society of Pediatrics, Chinese Medical Association; Editorial Board, Chinese Journal of Pediatrics; China Medicine Education Association Committee on Pediatrics. [Guidelines for the management of community-acquired pneumonia in children (2024 revision)]. Zhonghua Er Ke Za Zhi. 2024;62(10):920-930. doi:10.3760/cma.j.cn112140-20240728-00523

[3] Emeriaud G, López-Fernández YM, Iyer NP, Bembea MM, Agulnik A, Barbaro RP, et al. Executive Summary of the Second International Guidelines for the Diagnosis and Management of Pediatric Acute Respiratory Distress Syndrome (PALICC-2). Pediatr Crit Care Med. 2023;24(2):143-168. doi:10.1097/PCC.0000000000003147
