# Supplementary figures and images for: Soluble ST2 as a biomarker for predicting severe adverse events among pediatric patients with Mycoplasma pneumoniae pneumonia
Source: PLoS One. 2026 Apr 17;21(4):e0347651. doi: 10.1371/journal.pone.0347651 (PMC13089705; doi:10.1371/journal.pone.0347651)

S1 Fig. Flow diagram of study election process

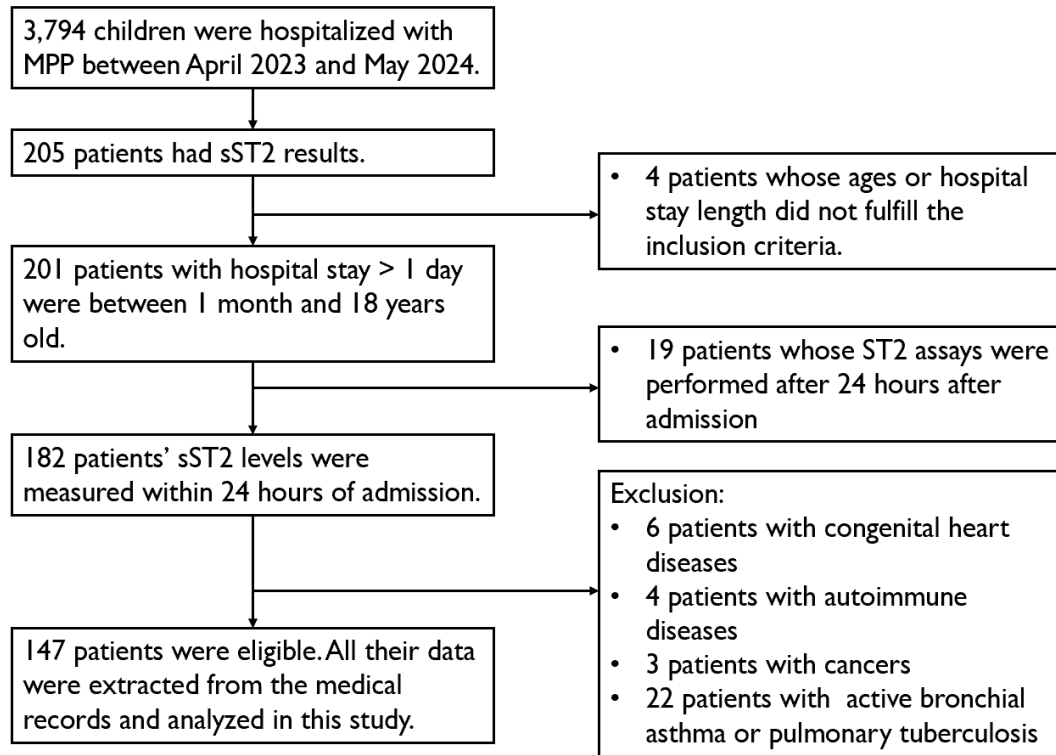

Supplement: S1 Fig — (PDF) [file pone.0347651.s002.pdf]

S2 Fig. Associations of ST2 with other biomarkers

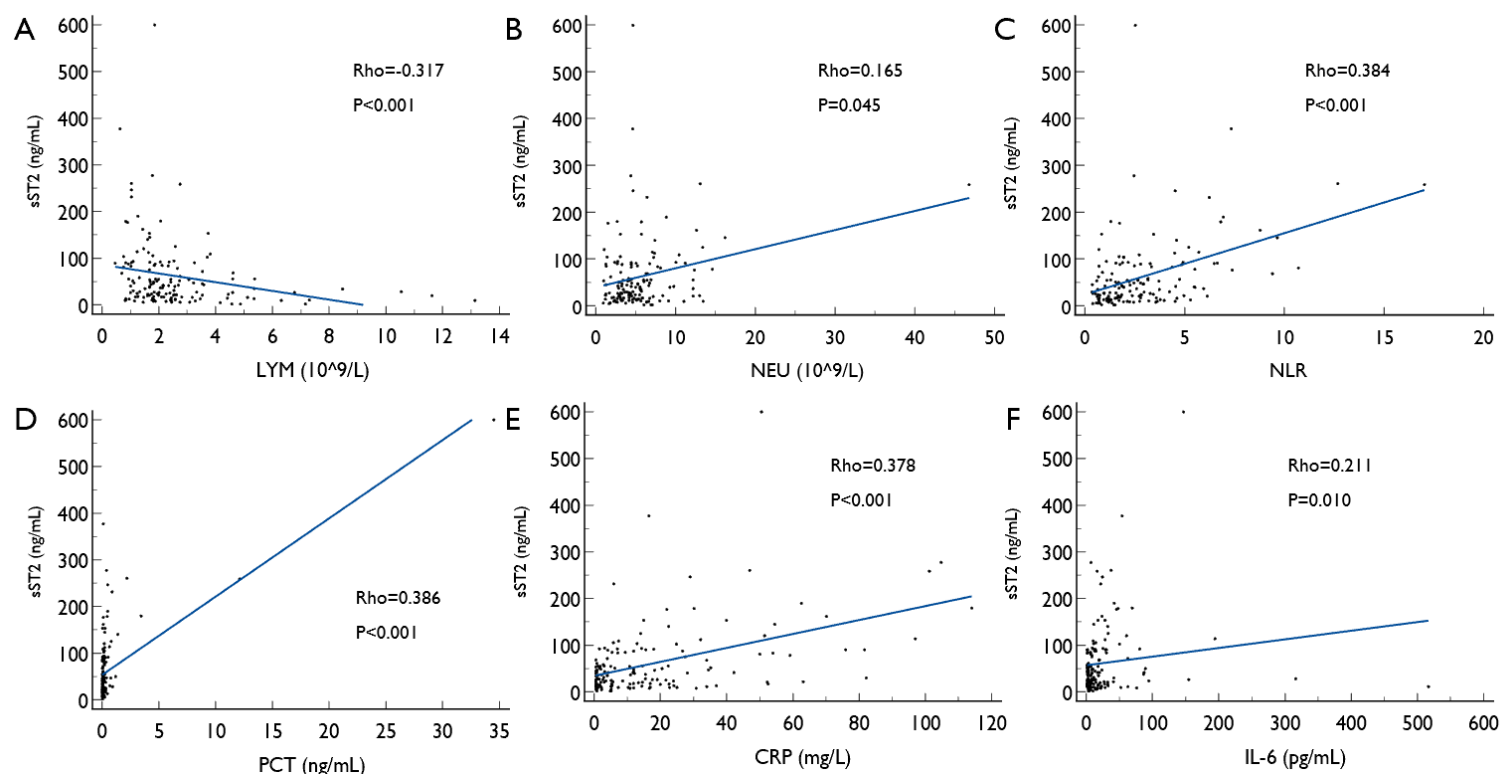

Supplement: S2 Fig — (PDF) [file pone.0347651.s003.pdf]

S3 Fig. Associations of ST2 with days of hospital stay and days of pre-admission fever

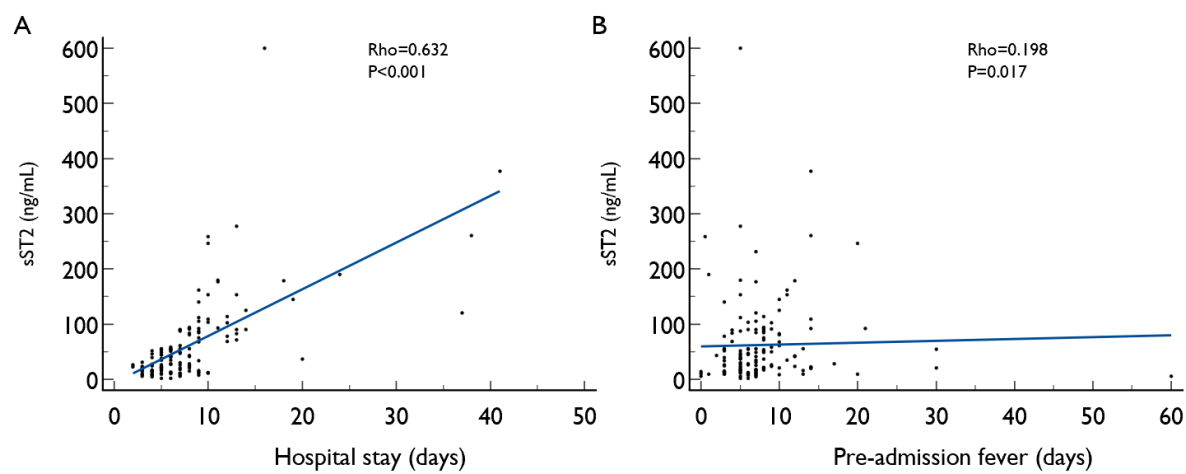

Supplement: S3 Fig — (PDF) [file pone.0347651.s004.pdf]

S4 Fig. Comparison of ST2 levels in patients with and without RMPP/MRMP (P=0.379)

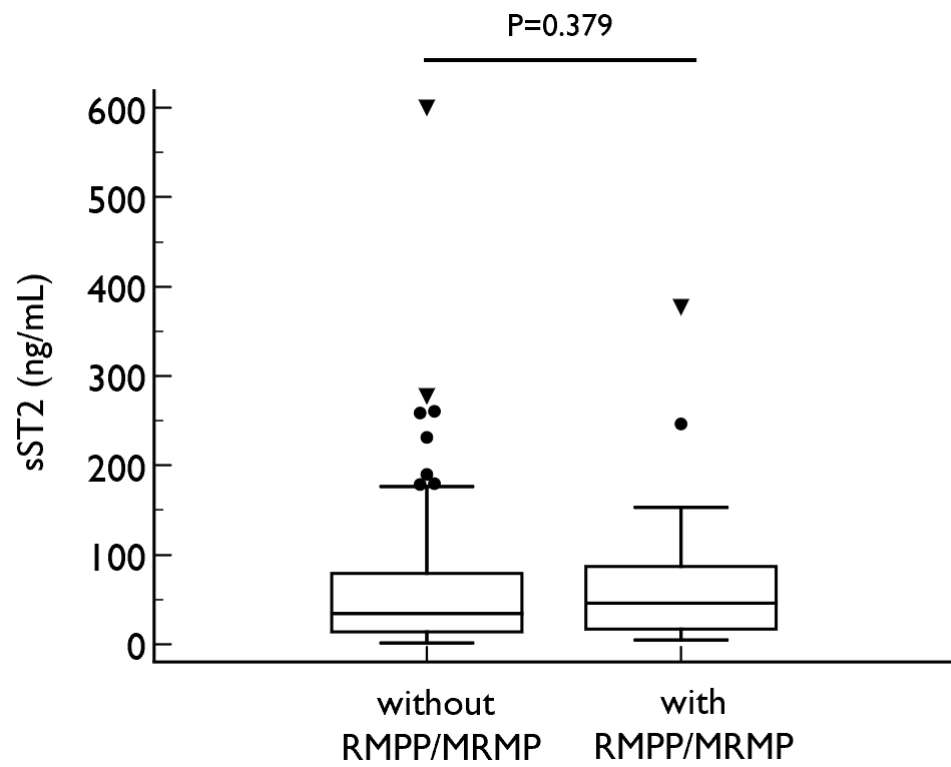

Supplement: S4 Fig — (PDF) [file pone.0347651.s005.pdf]
