# Supplementary material for: Soluble ST2 as a biomarker for predicting severe adverse events among pediatric patients with Mycoplasma pneumoniae pneumonia
Source: PLoS One. 2026 Apr 17;21(4):e0347651. doi: 10.1371/journal.pone.0347651 (PMC13089705; doi:10.1371/journal.pone.0347651)
Supplement: Attachment — Submitted filename: PONE-D-25-53987.pdf [file pone.0347651.s007.pdf]

PLOS ONE

Soluble ST2 is a potential biomarker for risk stratification of pediatric patients with mycoplasma pneumoniae pneumonia

--Manuscript Draft--

**Manuscript Number:**	PONE-D-25-53987
**Article Type:**	Research Article
**Full Title:**	Soluble ST2 is a potential biomarker for risk stratification of pediatric patients with mycoplasma pneumoniae pneumonia
**Short Title:**	Soluble ST2 is risk stratification of pediatric patients
**Corresponding Author:**	ZHICHENG YE<br>Shanghai Medical University Children's Hospital: Children's Hospital of Fudan University<br>Shanghai, CHINA
**Keywords:**	Mycoplasma pneumoniae; Children; pneumonia; ST2; Prognosis
**Abstract:**	Aim Mycoplasma pneumoniae (MP) is a leading cause of pneumonia in children. Early identification of severe cases is critical for improving outcomes. This study aimed to evaluate the association of soluble ST2 (sST2) with disease severity and in-hospital adverse events in pediatric MP pneumonia (MPP) for risk stratification.Methods We retrospectively analyzed 147 children with MPP admitted to the Children's Hospital of Fudan University, Shanghai, China, between June 2023 and December 2024. Demographic, clinical, and laboratory data were collected, including sST2, inflammatory markers (CRP, PCT, IL-6), and blood cell counts. Severe adverse events were defined as in-hospital death, ICU admission, diagnosis of sepsis or use of extracorporeal membrane oxygenation. Results sST2 levels were significantly higher in severe MPP cases and those with co-infections. Admission sST2 correlated positively with hospital stay and fever duration. Patients with pulmonary complications and/or expulmonary complications during hospital stay had significantly higher sST2 levels compared to those without complications. sST2 levels correlated positively with neutrophil count, neutrophil to lymphocyte ratio, PCT, CRP and IL-6 and negatively with lymphocyte count. Twelve patients experienced severe adverse events and had significantly higher sST2. ROC analysis showed that sST2 effectively predicted severe adverse events (AUC = 0.944, 95% CI 0.894-0.975, P<0.001), with an optimal cut-off of 114.18 ng/mL (sensitivity 91.7%, specificity 94.8%). The association remained significant after adjusting for age, gender, PCT, and IL-6.Conclusions sST2 was associated with disease severity and it predicted in-hospital adverse events more accurately than other inflammatory biomarkers, suggesting its potential as a prognostic biomarker for risk stratification in children with MPP.
**Order of Authors:**	ZHICHENG YE
	Jin Xu
	Fangying Cheng
	Tingting Li
	Lei Zhang
	Menghua Xu
	Luxi Chen
**Additional Information:**	
**Question**	**Response**
**Financial Disclosure**<br><br>Enter a financial disclosure statement that describes the sources of funding for the	The author(s) received no specific funding for this work.

work included in this submission. Review the submission guidelines for detailed requirements. View published research articles from *PLOS ONE* for specific examples.<br><br>This statement is required for submission and **will appear in the published article** if the submission is accepted. Please make sure it is accurate.<br><br>**Funded studies**<br>Enter a statement with the following details:<br>• Initials of the authors who received each award<br>• Grant numbers awarded to each author<br>• The full name of each funder<br>• URL of each funder website<br>• Did the sponsors or funders play any role in the study design, data collection and analysis, decision to publish, or preparation of the manuscript?<br><br>Did you receive funding for this work?	
**Competing Interests**<br><br>Use the instructions below to enter a competing interest statement for this submission. On behalf of all authors, disclose any competing interests that could be perceived to bias this work—acknowledging all financial support and any other relevant financial or non-financial competing interests.<br><br>This statement is required for submission and **will appear in the published article** if the submission is accepted. Please make sure it is accurate and that any funding sources listed in your Funding Information later in the submission form are also declared in your Financial Disclosure statement.<br><br>View published research articles from *PLOS ONE* for specific examples.	The authors have declared that no competing interests exist.

Powered by Editorial Manager® and ProduXion Manager® from Aries Systems Corporation

**NO authors have competing interests**<br><br>Enter: *The authors have declared that no competing interests exist.*<br><br>**Authors with competing interests**<br><br>Enter competing interest details beginning with this statement:<br><br>*I have read the journal's policy and the authors of this manuscript have the following competing interests: [insert competing interests here]*<br><br>* typeset	
**Ethics Statement**<br><br>Enter an ethics statement for this submission. This statement is required if the study involved:<br><br>• Human participants<br>• Human specimens or tissue<br>• Vertebrate animals or cephalopods<br>• Vertebrate embryos or tissues<br>• Field research<br><br>Write "N/A" if the submission does not require an ethics statement.<br><br>General guidance is provided below. Consult the submission guidelines for detailed instructions. **Make sure that all information entered here is included in the Methods section of the manuscript.**	Human specimens or tissue

Powered by Editorial Manager® and ProduXion Manager® from Aries Systems Corporation

**Format for specific study types**<br><br>**Human Subject Research (involving human participants and/or tissue)**<br>• Give the name of the institutional review board or ethics committee that approved the study<br>• Include the approval number and/or a statement indicating approval of this research<br>• Indicate the form of consent obtained (written/oral) or the reason that consent was not obtained (e.g. the data were analyzed anonymously)<br><br>**Animal Research (involving vertebrate animals, embryos or tissues)**<br>• Provide the name of the Institutional Animal Care and Use Committee (IACUC) or other relevant ethics board that reviewed the study protocol, and indicate whether they approved this research or granted a formal waiver of ethical approval<br>• Include an approval number if one was obtained<br>• If the study involved *non-human primates*, add *additional details* about animal welfare and steps taken to ameliorate suffering<br>• If anesthesia, euthanasia, or any kind of animal sacrifice is part of the study, include briefly which substances and/or methods were applied<br><br>**Field Research**<br><br>Include the following details if this study involves the collection of plant, animal, or other materials from a natural setting:<br>• Field permit number<br>• Name of the institution or relevant body that granted permission	
**Data Availability**<br><br>Authors are required to make all data underlying the findings described fully available, without restriction, and from the time of publication. PLOS allows rare exceptions to address legal and ethical concerns. See the PLOS Data Policy and FAQ for detailed information.	Yes - all data are fully available without restriction

Powered by Editorial Manager® and ProduXion Manager® from Aries Systems Corporation

A Data Availability Statement describing where the data can be found is required at submission. Your answers to this question constitute the Data Availability Statement and **will be published in the article**, if accepted.<br><br>**Important:** Stating 'data available on request from the author' is not sufficient. If your data are only available upon request, select 'No' for the first question and explain your exceptional situation in the text box.<br><br>Do the authors confirm that all data underlying the findings described in their manuscript are fully available without restriction?	
**Describe where the data may be found in full sentences. If you are copying our sample text, replace any instances of XXX with the appropriate details.**<br><br>• If the data are **held or will be held in a public repository**, include URLs, accession numbers or DOIs. If this information will only be available after acceptance, indicate this by ticking the box below. For example: *All XXX files are available from the XXX database (accession number(s) XXX, XXX.).*<br>• If the data are all contained **within the manuscript and/or Supporting Information files**, enter the following: *All relevant data are within the manuscript and its Supporting Information files.*<br>• If neither of these applies but you are able to provide **details of access elsewhere**, with or without limitations, please do so. For example:<br><br>*Data cannot be shared publicly because of [XXX]. Data are available from the XXX Institutional Data Access / Ethics Committee (contact via XXX) for researchers who meet the criteria for access to confidential data.*<br><br>*The data underlying the results presented in the study are available from (include the name of the third party*	All relevant data are within the manuscript and its Supporting Information files.

and contact information or URL).<br>• This text is appropriate if the data are owned by a third party and authors do not have permission to share the data.<br><br>* typeset	
Additional data availability information:	

Manuscript Click here to access/download;Manuscript;manuscript.docx

Soluble ST2 is a potential biomarker for risk stratification of pediatric patients with mycoplasma pneumoniae pneumonia

Fangying Cheng[1¶], Tingting Li[1¶], Lei Zhang[1], Menghua Xu[1], Luxi Chen[1], Zhicheng Ye[1]* and Jin Xu[1]*

[1]Department of Clinical Laboratory, Children's Hospital of Fudan University, National Children's Medical Center, Shanghai 201102, China

* Corresponding authors at

E-mail addresses:yzcrxj@163.com (Z. Ye), jinxu_125@163.com (J. Xu)

[¶]These authors contributed equally to this work.

Abstract

Aim *Mycoplasma pneumoniae* (MP) is a leading cause of pneumonia in children. Early identification of severe cases is critical for improving outcomes. This study aimed to evaluate the association of soluble ST2 (sST2) with disease severity and in-hospital adverse events in pediatric MP pneumonia (MPP) for risk stratification.**Methods** We retrospectively analyzed 147 children with MPP admitted to the Children's Hospital of Fudan University, Shanghai, China, between 01/04/2023 and 31/05/2024. Demographic, clinical, and laboratory data were collected, including sST2, inflammatory markers (CRP, PCT, IL-6), and blood cell counts. Severe adverse events were defined as in-hospital death, ICU admission, diagnosis of sepsis or use of extracorporeal membrane oxygenation. **Results** sST2 levels were significantly higher in severe MPP cases and those with co-infections. Admission sST2 correlated positively with hospital stay and fever duration. Patients with pulmonary complications and/or expulmonary complications during hospital stay had significantly higher sST2 levels compared to those without complications. sST2 levels correlated positively with neutrophil count, neutrophil to lymphocyte ratio, PCT, CRP and IL-6 and negatively with lymphocyte count. Twelve patients experienced severe adverse events and had significantly higher sST2. ROC analysis showed that sST2 effectively predicted severe adverse events (AUC = 0.944, 95% CI 0.894-0.975, $P<0.001$), with an optimal cut-off of 114.18 ng/mL (sensitivity 91.7%, specificity 94.8%). The association remained significant after adjusting for age, gender, PCT, and IL-6.**Conclusions** sST2 was associated with disease severity and it predicted in-hospital adverse events more accurately than other inflammatory biomarkers, suggesting its potential as a prognostic biomarker for risk stratification in children with MPP.

Key words: *Mycoplasma pneumoniae, children, pneumonia, ST2, prognosis*

Introduction

Mycoplasma pneumoniae (MP) is a major pathogen of community-acquired pneumonia (CAP) among children in China, especially those over 5 years old.[1] Mycoplasma pneumoniae pneumonia (MPP) typically presents with sore throat, coryza, intermittent irritating cough, accompanied by headache, fever and muscle pain. The infection occurs endemically with an epidemic peak every few years. After COVID-19 pandemic, China experienced a notable surge in MP infections from 2023 to 2024. This recent epidemic was characterized by an increased incidence of severe clinical manifestations and a high prevalence of macrolide-resistant M. pneumoniae (MRMP), complicating treatment.[2,3] These trends underscore the need for early identification of children at risk of severe or rapidly progressing disease to facilitate timely intervention.

Currently, physicians often rely on clinical gestalts, which refers to the ability of the physician to recognize patterns of disease to distinguish between severe and non severe diseases. Studies reported mixing findings on accuracy of this method to predict patients' outcome and inter-physician variations [4,5].Thus, clinical decision aids with subjective parameters are needed to aid in the identification of children at risk, yet existing tools show only moderate accuracy in pediatric respiratory infections and require further validation.[6-8] Blood-based biomarkers represent a promising alternative for assessing severity and predicting prognosis.

Soluble ST2 (sST2), a soluble form of the ST2 receptor, competes with membrane-bound ST2 for binding to interleukin-33 (IL-33), modulating inflammatory responses. [9] Immune dysregulation, including T-cell activation and cytokine-driven inflammation, contributes to MPP progression. [10,11] Although some adult studies link sST2 to prognosis in community-acquired pneumonia[12,13], its utility in pediatric MPP remains unclear.

Procalcitonin (PCT), C-reaction protein (CRP), interleukin 6 (IL-6) and immune cell counts such as white cell counts (WBC), neutrophil counts (NEU) and lymphocyte counts (LYM) are widely used laboratory tests for pneumonia patients. Studies have showed that CRP, IL-6 and neutrophil-to-lymphocyte ratios (NLR) associate with disease severity in pediatric MPP or predict refractory MPP (RMPP) [14-17]. Therefore, in this study, we aimed to evaluate the association of sST2 with in-hospital adverse events and clinical characteristics in pediatric MPP patients, and to assess its potential as a prognostic biomarker in comparison with conventional inflammatory markers.

Materials and methods

Subjects

This study retrospectively analyzed 147 children diagnosed with Mycoplasma pneumoniae pneumonia (MPP) who were admitted to the Children's Hospital of Fudan University in Shanghai, China, between 01/04/2023 and 31/05/2024. The retrospective analysis was conducted from 16/07/2024 to 31/01/2025. The inclusion criteria were: (1) MPP diagnosis, (2) age between 1 month and 18 years, (3) in-hospital stay > 24 hours, and (4)

available sST2 measurements at admission. The exclusion criteria were patients with (1) congenital heart diseases, (2) autoimmune diseases, (3) cancer, (4) active bronchial asthma or pulmonary tuberculosis, and (5) incomplete medical records.

In brief, patients were diagnosed with MPP if they met all the following criteria [2]: (1) had evidence of acute respiratory tract infections, accompanied by chest radiography-proven pneumonia; (2) a fourfold or greater increase in MP antibody titers of paired sera, or > 1:160 titer of single serum MP antibody, or MP-DNA(+) or MP-RNA(+). The diagnosis of SMPP is made in MPP patients with either of the following criteria [2]: (1) high fever (> 39 °C) for more than 5 days or fever for more than 7 days without a declining trend in peak temperature; (2) hypoxemia (maintained an SaO_2 < 92% on room air); (3) increasing respiratory and pulse rates with clinical evidence of respiratory distress and exhaustion with or without a raised $PaCO_2$; (4) signs of intrapulmonary infection, such as moderate to large pleural effusion, large area of pulmonary consolidation, plastic bronchitis, pulmonary embolism, necrotizing pneumonia, and acute asthma exacerbations; and (5) signs of extrapulmonary complications, such as meningoencephalitis, ascending (i.e., Guillain Barré) paralysis, myopericarditis, erythema multiforme, autoimmune hemolytic anemia, hemophagocytic syndrome, or disseminated intravascular coagulation.

This study was in accordance with the Declaration of Helsinki and approved by the Ethics Committee of the Children’s Hospital of Fudan University (Ethics No:2024-129).We confirm that all methods were performed in accordance with the relevant guidelines and regulations. This study is a retrospective analysis, only involving the statistical analysis of patient specimen data, and all patient information has been anonymized and does not contain

sensitive data, so the exemption from informed consent procedure is applied.

Clinical data collection

Data were obtained from medical records, including demographic characteristics, pathogens, pulmonary and expulmonary complications, and severe adverse events during hospitalization. Severe adverse events were defined as in-hospital death, ICU admission, diagnosis of sepsis or use of extracorporeal membrane oxygenation (ECMO). Pulmonary complications included acute respiratory distress syndrome, respiratory failure, pulmonary necrosis, pleural effusion, plastic bronchitis, pulmonary embolism and pulmonary atelectasis. Expulmonary complications included complications of nervous system, circulatory system, blood, skin and mucous, liver, kidney, muscles, blood pressure and pancreas.

Laboratory tests

Laboratory test results including sST2, CRP, PCT, IL-6, WBC, LYM, NEU and NEU to LYM ratio (NLR) were collected. sST2 and IL-6 were measured by fluorescent immunoassays on Pylon 3D immune analyzer from ETheathcare Inc. (Suzhou, China). PCT were measured by electrochemiluminescence assays using the instruments and reagents from Roche Diagnostics GmbH (Mannheim, Germany). CRP were measured by turbidimetric immunoassays using the instruments and reagents from Shanghai Upper Bio-Tech Pharma Ltd. (Shanghai, China). Complete blood counts were acquired by XN-20 analyzer using reagents from Sysmex Corp.(Kobe, Japan).

Statistical analysis

Continuous variables were presented as median (interquartile range) and compared by Mann-Whitney test or Kruskal-Wallis test. Categorical variables were expressed as numbers (%) and compared using Fisher's exact test. Spearman correlation was used for correlation analyses between sST2 and other parameters. The optimal cutoff for sST2 was determined by maximizing Youden's index from receiver operating characteristic (ROC) curve analysis. The areas under the curve (AUC) of ROCs were calculated to compare the predictive performance of biomarkers for severe adverse events. Multivariable logistic regression assessed the independent prognostic value of sST2 after adjustment for age and gender or other biomarkers. All statistical tests were two tailed, and a value of $P < 0.05$ was considered statistically significant. Statistical analyses were performed using MedCalc statistical software version 22.009 (MedCalc Ltd., Ostend, Belgium).

Results

Patient Characteristics

147 children (77 females, 52.38%) hospitalized with MPP were included, of whom the median age was 6 years and media hospital stay was 7 days. Severe adverse events occurred in 12 patients (8.2%), including 1 death, 10 ICU admissions, 5 patients diagnosed of sepsis and 2 patients needing ECOM during their hospital stay. The demographic data, clinical

parameters and laboratory results at admission of all the study subjects were presented were in Table 1 and compared between the groups with and without severe adverse events. The two groups showed similar gender distribution and fever duration before hospital admission. However, in the group with severe adverse events, patients were significantly younger and weighed lower. There were also significantly higher portion of severe MPP patients and patients with pulmonary and expulmonary complications in the group of severe adverse events, although the portions of RMPP/MRMP and co-infection with other pathogens were similar between the two groups. The group with severe adverse events stayed significantly longer in hospital.

Table 1 Patient characteristics

		Severe Adverse Events		
**Characteristic**	Overall[1] (N = 147)	No (N = 135)[1]	Yes (N = 12)[1]	**p-value**[2]
Gender				0.135
Female	77.00 (52.38%)	68.00 (50.37%)	9.00 (75.00%)	
Male	70.00 (47.62%)	67.00 (49.63%)	3.00 (25.00%)	
Age	6.00 (4.00, 9.00)	6.00 (4.00, 9.00)	4.00 (1.75, 6.00)	0.047
Weight (kg)	23.00 (16.50, 31.00)	23.20 (17.00, 31.00)	15.50 (12.75, 20.00)	0.018
Severe MP	46.00 (31.29%)	34.00 (25.19%)	12.00 (100.00%)	<0.001
RMPP/MRMP	51.00 (34.69%)	47.00 (34.81%)	4.00 (33.33%)	0.999
Co-infections	83.00 (56.46%)	74.00 (54.81%)	9.00 (75.00%)	0.231

Bacteria infection	21.00 (14.29%)	18.00 (13.33%)	3.00 (25.00%)	0.38
Virus infection	72.00 (48.98%)	63.00 (46.67%)	9.00 (75.00%)	0.074
Other infection	5.00 (3.40%)	3.00 (2.22%)	2.00 (16.67%)	0.053
Severe adverse events				
Death	1.00 (0.68%)	0.00 (0.00%)	1.00 (8.33%)	
ICU	10.00 (6.80%)	0.00 (0.00%)	10.00 (83.33%)	
Sepsis	5.00 (3.40%)	0.00 (0.00%)	5.00 (41.67%)	
ECMO	2.00 (1.36%)	0.00 (0.00%)	2.00 (16.67%)	
Complications				<0.001
None	99.00 (67.35%)	99.00 (73.33%)	0.00 (0.00%)	
Expulmonary	14.00 (9.52%)	14.00 (10.37%)	0.00 (0.00%)	
Pulmonary	24.00 (16.33%)	19.00 (14.07%)	5.00 (41.67%)	
Pulmonary and expulmonary	10.00 (6.80%)	3.00 (2.22%)	7.00 (58.33%)	
Pulmonary complications	34.00 (23.13%)	22.00 (16.30%)	12.00 (100.00%)	<0.001
ARDS	2.00 (1.36%)	0.00 (0.00%)	2.00 (16.67%)	0.006
Respiratory failure	11.00 (7.48%)	2.00 (1.48%)	9.00 (75.00%)	<0.001
Pulmonary necrosis	3.00 (2.04%)	1.00 (0.74%)	2.00 (16.67%)	0.018

Pleural effusion	11.00 (7.48%)	9.00 (6.67%)	2.00 (16.67%)	0.222
Plastic bronchitis	2.00 (1.36%)	2.00 (1.48%)	0.00 (0.00%)	0.999
Pulmonary embolism	1.00 (0.68%)	1.00 (0.74%)	0.00 (0.00%)	0.999
Pulmonary consolidation	54.00 (36.73%)	52.00 (38.52%)	2.00 (16.67%)	0.211
Pulmonary atelectasis	11.00 (7.48%)	10.00 (7.41%)	1.00 (8.33%)	0.999
Extrapulmonary complications	24.00 (16.33%)	17.00 (12.59%)	7.00 (58.33%)	<0.001
Nervous system	4.00 (2.72%)	1.00 (0.74%)	3.00 (25.00%)	0.002
Circulatory system	10.00 (6.80%)	7.00 (5.19%)	3.00 (25.00%)	0.036
Blood system	5.00 (3.40%)	3.00 (2.22%)	2.00 (16.67%)	0.053
Skin and mucous	5.00 (3.40%)	5.00 (3.70%)	0.00 (0.00%)	0.999
Other systems[3]	5.00 (3.40%)	2.00 (1.48%)	3.00 (25.00%)	0.004
Fever duration before hospital admission (days)	7.00 (5.00, 9.00)	7.00 (5.00, 9.00)	8.00 (5.00, 10.50)	0.415
Hospital stay (days)	7.00 (5.00, 9.00)	6.00 (5.00, 9.00)	17.50 (11.00, 37.00)	<0.001

ST2 (ng/mL)	39.26 (16.51, 84.64)	34.03 (15.69, 67.99)	170.69 (132.53, 246.26)	<0.001
WBC (10^9/L)	7.52 (5.59, 10.56)	7.52 (5.59, 10.36)	8.95 (5.34, 15.10)	0.408
NEU (10^9/L)	4.59 (3.07, 6.60)	4.46 (3.07, 6.39)	6.92 (3.76, 12.87)	0.073
LYM (10^9/L)	2.00 (1.47, 2.81)	2.11 (1.61, 3.18)	1.54 (1.15, 1.78)	0.01
NLR	2.13 (1.24, 3.69)	1.95 (1.20, 3.41)	5.72 (2.11, 8.07)	0.004
CRP (mg/L)	10.88 (2.26, 24.00)	7.44 (2.00, 22.01)	48.75 (18.51, 58.58)	<0.001
PCT (ng/mL)	0.09 (0.04, 0.20)	0.08 (0.04, 0.17)	0.85 (0.23, 1.80)	<0.001
IL-6 (pg/mL)	10.99 (4.20, 27.36)	9.66 (3.62, 21.60)	40.10 (28.28, 65.07)	<0.001

[1]n (%); Median (Q1, Q3) [2]Fisher's exact test; Mann-Whitney test [3]Complications in liver, kidney, muscles, blood pressure, or pancreas

Correlation between sST2 and other laboratory biomarkers and clinical parameters

sST2 levels correlated positively with NEU (rho=0.165, P=0.045), NLR (rho=0.384, P<0.001), PCT (rho=0.386, P<0.001), CRP (rho=0.378, P<0.001) and IL-6 (rho=0.211, P =0.010) and negatively with LYM (rho=-0.317, P<0.001). (Figure S1) We also found sST2 at admission positively correlated with length of hospital stay (rho=0.632, P<0.001) and pre-admission fever duration (rho=0.198, P=0.017). (Figure S2)

Severe MPP patients and MPP patients co-infected with other respiratory track pathogens had significantly higher sST2 levels (71.91 [30.76-140.12] vs 26.2 [15.15-54.69], P<0.001; 48.46 [23.23-94.17] vs 23.47 [13.18-52.87] ng/mL, P<0.001, respectively). (Figure 1) . However, patients with or without RMPP/MRMP had similar sST2 levels (45.97 [17.00-87.01] vs 34.39 [13.90-79.26] ng/mL, P=0.379).(Figure S3) We also found patients with pulmonary complications and/or expulmonary complications during hospital stay had significantly higher sST2 levels at admission compared to those without complications (77.84 [29.56-147.16] vs 26.68 [14.53-54.54] ng/mL, P<0.001). (Figure 3A) Pairwise comparison showed that patients with both pulmonary and expulmonary complications had highest sST2 (170.15 [140.12– 245.95] ng/mL) followed by those with only pulmonary complications (58.80 [28.79– 91.93] ng/mL), while sST2 levels were similar in those without complications (26.68 [14.53- 54.54] ng/mL) and patients with only expulmonary complications (41.78 [14.32-100.05] ng/mL). (Figure 3B)

Figure S1 Associations of ST2 with other biomarkers

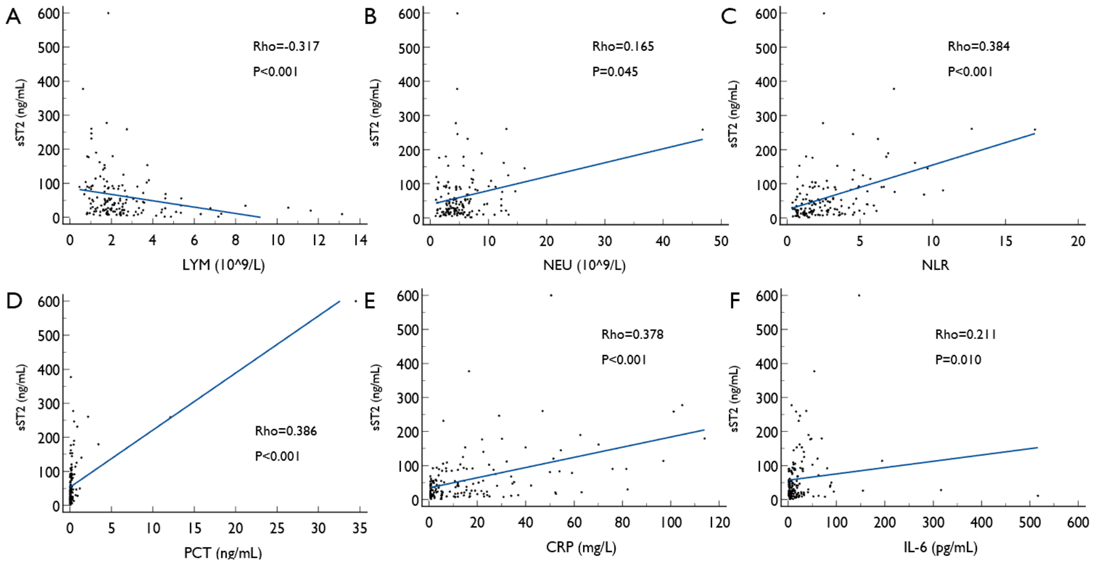

Figure S2 Associations of ST2 with days of hospital stay and days of pre-admission fever

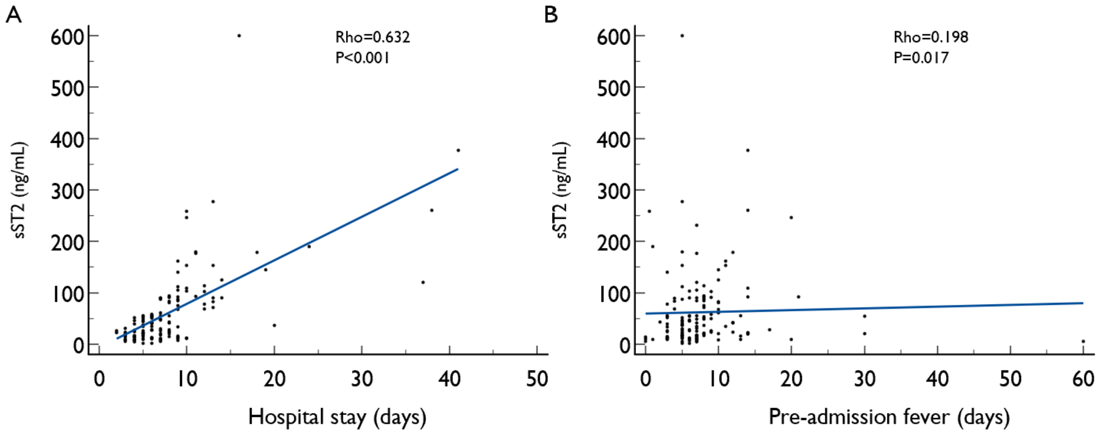

Figure 1 Comparison of ST2 levels in patients of different severity and different pathogens

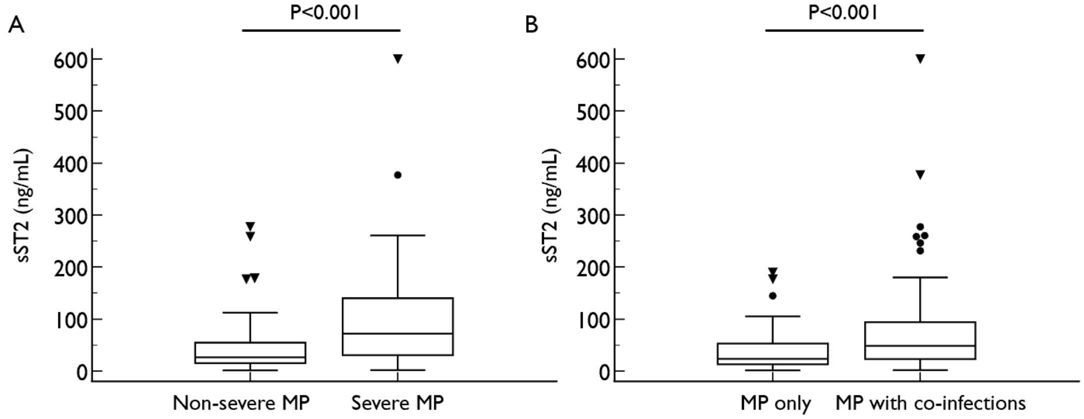

Figure S3 Comparison of ST2 levels in patients with and without RMPP/MRMP (P=0.379)

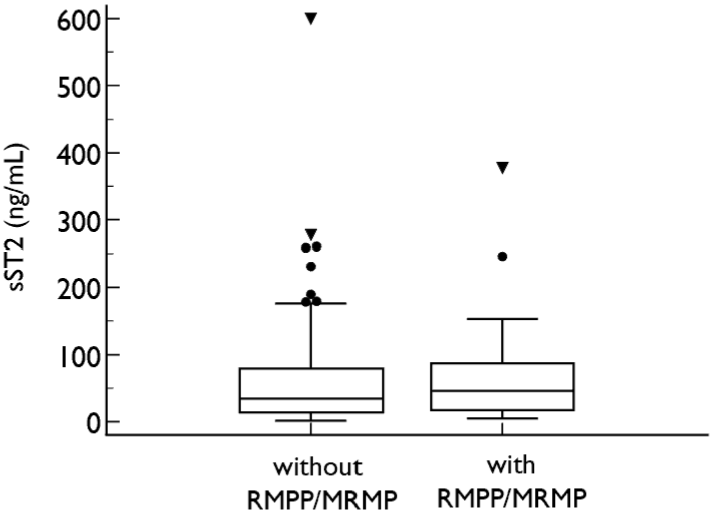

Figure 2 Comparison of sST2 levels in patients with and without complications (A) and different types of complications (B)

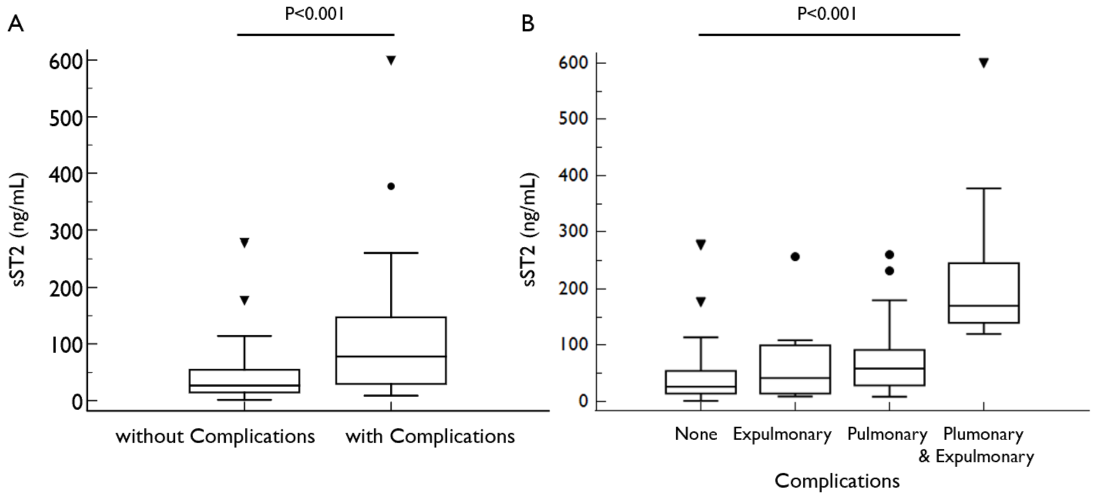

Associations of sST2 with severe adverse events during hospital stay

The group with severe adverse events showed significantly higher sST2 compared to the group without severe adverse events (170.69 [132.53, 246.26] vs 34.03 [15.69, 67.99] ng/mL, $P<0.001$) (Figure 3A). These patients also had higher inflammatory biomarkers (CRP, PCT and IL-6), lower LYM and higher NLR (all $P<0.05$, Table 1). Meanwhile, WBC and NEU did not differ significantly in two groups.

ROC analysis showed that sST2 effectively predicted patients with severe adverse events (AUC = 0.944, 95% CI 0.894-0.975, $P<0.001$), with an optimal cut-off of 114.18 ng/mL (sensitivity 91.7%, specificity 94.8%) determined by Youden's index. According to ROC AUCs, sST2 showed slightly greater predictive accuracy than PCT (AUC: 0.944 vs 0.900, P=0.427) and IL-6 (AUC: 0.944 vs 0.858, P=0.101) without statistically significant difference, but sST2 showed significantly higher predictability compared to CRP (AUC: 0.944 vs 0.822, P=0.027), LYM (AUC: 0.944 vs 0.724, $P<0.001$) and NLR (AUC: 0.944 vs 0.750, P=0.025). (Figure 3B) Logistic regression analysis confirmed that the increase of sST2 was associated with increased risk of severe adverse events (unadjusted OR= 1.023 per unit increase, 95% CI 1.012-1.033, $P<0.001$). The association remained significant after adjusting for age and gender (adjusted OR= 1.023, 95% CI 1.022-1.034, $P<0.001$) or PCT and IL-6 (adjusted OR= 1.025, 95% CI 1.013 to 1.037, $P<0.001$) (Table 3).

Figure 3 Comparison of sST2 levels in patients with and without severe adverse events and Comparison of ROC curves of different biomarkers in differentiating patients with severe adverse events

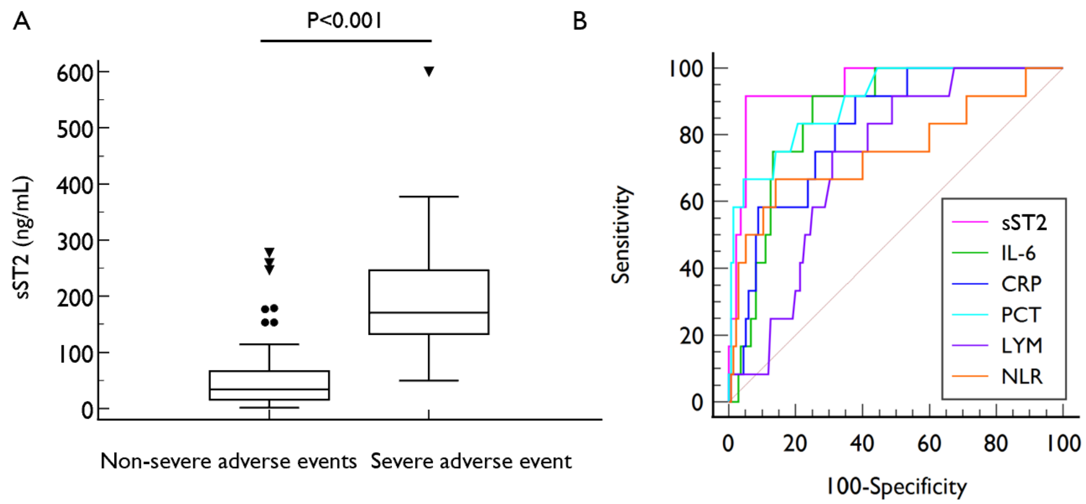

Table 2 Multivariant logistic regression of ST2 on prediction of severe adverse events

	Odds ratio	95% CI	P
Single variant model			
sST2	1.023	1.012 to 1.033	<0.001
Multivariant model 1			
sST2	1.023	1.012 to 1.034	<0.001
Age	0.896	0.697 to 1.152	0.371
Gender="M"	0.346	0.059 to 2.040	0.452
Multivariant model 2			
sST2	1.025	1.013 to 1.037	<0.001
IL-6	1.006	0.997 to 1.016	0.181

PCT	0.869	0.607 to 1.246	0.445

Discussion

The prolonged implementation of non pharmaceutical interventions to tackle Covid-19 pandemic between 2020 and 2022 led to a significant “immunity debt”. Consequently, MP infection undergone a prolonged non-seasonal epidemic, which emerged in April 2023 and reached its peak around October before finally subsided by June 2024 across mainland China. This epidemic affected a vast majority of the children, especially infants and preschool children, who have not been exposed to MP and resulted in increased incidences of MPP.[18] The high prevalence of macrolide-resistant MP (MRMP) further complicated treatment, highlighting the need for reliable risk stratification tools to guide intensive therapy in high-risk cases. In this retrospective study, we found higher sST2 was associated with severe MPP, co-infections, and complications, indicating its use in assessing disease severity. More importantly, patients who experienced severe adverse events during hospital stay had significantly higher sST2 level at admission, and ROC analysis showed that sST2 effectively predicted these events (AUC = 0.944), and its prognostic value remained significant after adjusting for age, gender, or other biomarkers. These findings support the potential of sST2 as a prognosis biomarker for risk stratification in pediatric MPP patients.

A previous study in pediatric CAP patients found limited utility of WBC, NEU, CRP, and PCT in discriminating non-severe from severe disease in children, though CRP and PCT may have some use in predicting the most severe outcomes.[19] However, only 7.3% of cases in

that study involved MPP. The levels of inflammatory biomarkers varied between CAP of different pathogens [20]. Studies in MPP found that LDH had a clinical value in predicting Mycoplasma pneumoniae necrotizing pneumonia [21], and NLR and CRP were independent risk factors for severe MPP in children [22]. We found sST2 positively correlated with other inflammatory biomarkers and demonstrated sST2 had superior predictive performance than CRP and NLR in predicting severe adverse events. In adult CAP, sST2 has been shown to predict clinical stability, in-hospital mortality, and ICU admission in CAP patients, improving the prediction power of CURB-65 or PSI scores.[12,13] Recently data in adults also suggested that sST2 as a dual cardio-inflammatory biomarker was a valuable prognostic factor in COVID-19 for it related to both myocardial injury and severity of inflammation. [23] To our knowledge, this is the first study to establish sST2 as a predictor of short-term prognosis in pediatric MPP.

sST2 is a decoy receptor of IL-33 and IL-33/ST2 axis plays a role in immune regulation.[24] Excessive immune activation is thought to drive severe pulmonary and extrapulmonary manifestations in MPP. [18] IL-33 binding to membrane-bound ST2 activates the MyD88/NF-κB signaling pathway to enhance Th2 response. Notably, Th1 and Th17 cells both express sST2 and it was suggested that increased sST2 production promotes the Th1/Th17 response and reduced the ST2-mediated Th2/Treg response through sequestering IL-33 [25]. In addition, in vitro study showed proinflammatory cytokines such as IL-1β and TNF-α enhance sST2 production in human lung epithelial cells in vitro. [24] These mechanisms may explain the link between high sST2 level, immune dysregulation, and disease severity and progression to adverse outcome in MPP. Further, the association of

sST2 and severe adverse events also indicated that IL-33/ST2 axis is a potential therapeutic target for MPP.

There are limitations of this study. First, the sample size was relatively small, with only 12 severe adverse events, limiting the ability to perform multivariable adjustments for multiple confounders. simultaneously. Second, clinical parameters such as pediatric early warning scores or oxygenation indices were not available for comparison. In the future, we plan to conduct a multi-center prospective study with a larger study population and comprehensive clinical data to validate the clinical value of sST2 in children with pneumonia.

Conclusions

In this retrospective study of pediatric MPP patients, we found sST2's association with disease severity and it can predict in-hospital adverse events with more accurate than other inflammatory biomarkers, which suggested its potential as a prognostic biomarker to aid management of children with MPP.

References

1. National Health Commission of the People's Republic of China. Guidelines for diagnosis and treatment of Mycoplasma pneumonae pneumonia in children (2023 Edition). *Chin J Rational Drug Use*. 2023;20(3):16-24. doi: 10.3969/j.issn.2096-3327.2023.03.003

2. Zhang XB, He W, Gui YH, et al. Current Mycoplasma pneumoniae epidemic among

children in Shanghai: unusual pneumonia caused by usual pathogen. *World J Pediatr*. 2024;20(1):5-10. doi:10.1007/s12519-023-00793-9

3. Chen Y, Li X, Fu Y, Yu Y, Zhou H. Whole-genome sequencing unveils the outbreak of Mycoplasma pneumoniae in mainland China. *Lancet Microbe*. 2024;5(9):100870. doi:10.1016/S2666-5247(24)00086-7

4. Gao HM, Ambroggio L, Shah SS, Ruddy RM, Florin TA. Predictive value of clinician "gestalt" in pediatric community-acquired pneumonia. *Pediatrics*. 2021;147(5):e2020041582. doi:10.1542/peds.2020-041582

5. Rees CA, Kisenge R, Ideh RC, et al. Predictive value of clinician impression for readmission and postdischarge mortality among neonates and young children in Dar es Salaam, Tanzania and Monrovia, Liberia. *BMJ Paediatr Open*. 2023;7(1):e001972. doi:10.1136/bmjpo-2023-001972

6. Ambroggio L, Brokamp C, Mantyla R, et al. Validation of the British Thoracic Society Severity Criteria for Pediatric Community-acquired Pneumonia. *Pediatr Infect Dis J*. 2019;38(9):894-899. doi:10.1097/INF.0000000000002380

7. Florin TA, Brokamp C, Mantyla R, et al. Validation of the Pediatric Infectious Diseases Society-Infectious Diseases Society of America Severity Criteria in Children With Community-Acquired Pneumonia. *Clin Infect Dis*. 2018;67(1):112-119. doi:10.1093/cid/ciy031

8. Florin TA, Ambroggio L, Lorenz D, et al. Development and internal validation of a prediction model to risk stratify children with suspected community-acquired pneumonia. *Clin Infect Dis*. 2021;73(9):e2713-e2721. doi:10.1093/cid/ciaa1690

9. Zhou Y, Xu Z, Liu Z. Role of IL-33-ST2 pathway in regulating inflammation: current

evidence and future perspectives. *J Transl Med*. 2023;21(1):902. doi:10.1186/s12967-023-04782-4

10. Zhu Y, Luo Y, Li L, et al. Immune response plays a role in Mycoplasma pneumoniae pneumonia. *Front Immunol*. 2023;14:1189647. doi:10.3389/fimmu.2023.1189647

11. Gao M, Wang K, Yang M, et al. Transcriptome analysis of bronchoalveolar lavage fluid from children with Mycoplasma pneumoniae pneumonia reveals natural killer and T cell-proliferation responses. *Front Immunol*. 2018;9:1403. doi:10.3389/fimmu.2018.01403

12. Watanabe M, Takizawa H, Tamura M, et al. Soluble ST2 as a prognostic marker in community-acquired pneumonia. *J Infect*. 2015;70(5):474-482. doi:10.1016/j.jinf.2015.02.004

13. Zhang T, Zeng Y, Lin R, et al. Incorporation of suppression of tumorigenicity 2 into random survival forests for enhancing prediction of short-term prognosis in community-acquired pneumonia. *J Clin Med*. 2022;11(20):6015. doi:10.3390/jcm11206015

14. Zhang Y, Zhou Y, Li S, Yang D, Wu X, Chen Z. The clinical characteristics and predictors of refractory Mycoplasma pneumoniae pneumonia in children. *PLoS One*. 2016;11(5):e0156465. doi:10.1371/journal.pone.0156465

15. Fan F, Lv J, Yang Q, Jiang F. Clinical characteristics and serum inflammatory markers of community-acquired mycoplasma pneumonia in children. *Clin Respir J*. 2023;17(7):607-617. doi:10.1111/crj.13620

16. Ling Y, Ning J, Xu Y. Explore the predictive value of peripheral blood cell parameters in refractory Mycoplasma pneumoniae pneumonia in children over 6 years old. *Front Pediatr*. 2021;9:659677. doi:10.3389/fped.2021.659677

17. Nagoba BS, Dhotre SV, Gavkare AM, Mumbre SS, Dhotre PS. Understanding serum

inflammatory markers in pediatric Mycoplasma pneumoniae pneumonia. *World J Clin Pediatr*. 2024;13(4):98809. doi:10.5409/wjcp.v13.i4.98809

18. Ding G, Zhang X, Vinturache A, van Rossum AMC, Yin Y, Zhang Y. Challenges in the treatment of pediatric Mycoplasma pneumoniae pneumonia. *Eur J Pediatr*. 2024;183(7):3001-3011. doi:10.1007/s00431-024-05519-1
19. Florin TA, Ambroggio L, Brokamp C, et al. Biomarkers and disease severity in children with community-acquired pneumonia. *Pediatrics*. 2020;145(6):e20193728. doi:10.1542/peds.2019-3728
20. Guo H, Liang J, Lin H, et al. Differentiate clinical characteristics between viral pneumonia and Mycoplasma pneumoniae and nomograms for predicting Mycoplasma pneumoniae: a retrospective study in primary hospitals. *Pediatr Infect Dis J*. 2023;42(12):1035-1040. doi:10.1097/INF.0000000000004082
21. Yanhong R, Shuai Z, Dan C, Xiaomin S. Predictive value of lactate dehydrogenase for Mycoplasma pneumoniae necrotizing pneumonia in children based on decision curve analysis and dose-response analysis. *Sci Rep*. 2024;14(1):9803. doi:10.1038/s41598-024-60359-1
22. Zhang X, Sun R, Jia W, Li P, Song C. A new dynamic nomogram for predicting the risk of severe Mycoplasma pneumoniae pneumonia in children. *Sci Rep*. 2024;14(1):8260. doi:10.1038/s41598-024-58784-3
23. Miftode RS, Petriș AO, Onofrei Aursulesei V, et al. The novel perspectives opened by ST2 in the pandemic: a review of its role in the diagnosis and prognosis of patients with heart failure and COVID-19. *Diagnostics (Basel)*. 2021;11(2):175. doi:10.3390/diagnostics11020175
24. Griesenauer B, Paczesny S. The ST2/IL-33 axis in immune cells during inflammatory

diseases. *Front Immunol*. 2017;8:475. doi:10.3389/fimmu.2017.00475

25. Zhang J, Ramadan AM, Griesenauer B, et al. ST2 blockade reduces sST2-producing T cells while maintaining protective mST2-expressing T cells during graft-versus-host disease. *Sci Transl Med*. 2015;7(308):308ra160. doi:10.1126/scitranslmed.aab0166

Click here to access/download
Supporting Information
Research programme.docx

Click here to access/download
Supporting Information
st2.pdf
