# Supplementary material for: Soluble ST2 as a biomarker for predicting severe adverse events among pediatric patients with Mycoplasma pneumoniae pneumonia
Source: PLoS One. 2026 Apr 17;21(4):e0347651. doi: 10.1371/journal.pone.0347651 (PMC13089705; doi:10.1371/journal.pone.0347651)
Supplement: Attachment — Submitted filename: 260325 Response to Reviewers.docx [file pone.0347651.s009.docx]

PONE-D-25-53987

Soluble ST2 is a potential biomarker for risk stratification of pediatric patients with mycoplasma pneumoniae pneumonia

PLOS One

Dear Editor and Reviewers,

Thank you very much for your valuable comments and constructive suggestions on our manuscript. We have carefully gone over all the feedback and revised the manuscript accordingly. The replies to all the comments are provided in the uploaded response document. A revised manuscript has been uploaded too.

We appreciate all your time and efforts in helping us make this study and this manuscript better.

We look forward to hearing from you.

Best regards,

Zhicheng Ye

The responses to the editor and the reviewers are as the below in blue.

- Journal Requirements:

When submitting your revision, we need you to address these additional requirements.

1. Please ensure that your manuscript meets PLOS ONE's style requirements, including those for file naming. The PLOS ONE style templates can be found at https://journals.plos.org/plosone/s/file?id=wjVg/PLOSOne_formatting_sample_main_body.pdf and https://journals.plos.org/plosone/s/file?id=ba62/PLOSOne_formatting_sample_title_authors_affiliations.pdf

We have reformatted the manuscript according to the two style templates.

2. PLOS requires an ORCID iD for the corresponding author in Editorial Manager on papers submitted after December 6th, 2016. Please ensure that you have an ORCID iD and that it is validated in Editorial Manager. To do this, go to ‘Update my Information’ (in the upper left-hand corner of the main menu), and click on the Fetch/Validate link next to the ORCID field. This will take you to the ORCID site and allow you to create a new iD or authenticate a pre-existing iD in Editorial Manager.

The ORCID iDs for the corresponding authors have been updated via submission system

（https://orcid.org/my-orcid?orcid=0009-0009-6967-6969，ORCID：0009-0009-6967-6969）.

3. Please provide additional details regarding participant consent. In the ethics statement in the Methods and online submission information, please ensure that you have specified what type you obtained (for instance, written or verbal, and if verbal, how it was documented and witnessed). If your study included minors, state whether you obtained consent from parents or guardians. If the need for consent was waived by the ethics committee, please include this information.

Once you have amended this/these statement(s) in the Methods section of the manuscript, please add the same text to the “Ethics Statement” field of the submission form (via “Edit Submission”).

For additional information about PLOS ONE ethical requirements for human subjects research, please refer to http://journals.plos.org/plosone/s/submission-guidelines#loc-human-subjects-research.

This study was approved by the Ethics Committee of the Children’s Hospital of Fudan University (Ethics No:2024-129), and need for consent was waived by the ethics committee. We have included an Ethics Statement subsection in the Materials and methods: “This study was conducted in accordance with the Declaration of Helsinki and was approved by the Ethics Committee of the Children’s Hospital of Fudan University (Ethics No. 2024-129). We confirm that all methods were performed in accordance with the relevant guidelines and regulations. This study is a retrospective analysis, only involving the statistical analysis of patient data, and all patient information has been anonymized and does not contain sensitive data; therefore, the requirement for informed consent was waived by the ethics committee.”

The same text has been added to the “Ethics Statement” field of the submission form via the submission system.

4. Please include your full ethics statement in the ‘Methods’ section of your manuscript file. In your statement, please include the full name of the IRB or ethics committee who approved or waived your study, as well as whether or not you obtained informed written or verbal consent. If consent was waived for your study, please include this information in your statement as well.

As described above, the full ethics statement has been provided in the ‘Materials and methods-Ethics Statement’ and via the online submission system.

5. We notice that your supplementary [figures] are included in the manuscript file. Please remove them and upload them with the file type 'Supporting Information'. Please ensure that each Supporting Information file has a legend listed in the manuscript after the references list.

The supplementary figures have been removed from the manuscript file and a list of supplementary figure captions has been added in the manuscript after the reference list. A supporting document named S1-4_Fig.pdf is uploaded via the submission system.

6. Please ensure that you refer to Figure 2 in your text as, if accepted, production will need this reference to link the reader to the figure.

It was a typo and we have corrected it. Figure 2 is now referred to in ‘Results- Correlation between sST2 and other laboratory biomarkers and clinical parameters’ section.

7. We note you have included a table to which you do not refer in the text of your manuscript. Please ensure that you refer to Table 2 in your text; if accepted, production will need this reference to link the reader to the Table.

It was a typo and we have corrected it. Table 2 is now referred to in ‘Results- Associations of sST2 with severe adverse events during hospital stay’ section.

8. Please include a copy of Table 3, which you refer to in your text on page 15.

It was a typo and it should be ‘Table 2’. We have corrected it.

9. Please include captions for your Supporting Information files at the end of your manuscript, and update any in-text citations to match accordingly. Please see our Supporting Information guidelines for more information: http://journals.plos.org/plosone/s/supporting-information.

A list of supplementary figure and file captions has been added after the reference list. The supplementary figures are cited as S1-S4 Fig in the manuscript. A supporting document containing these figures has been uploaded via the submission system (S1-4_Fig.pdf). Another supporting file (S1_File.pdf) has been added too.

10. If the reviewer comments include a recommendation to cite specific previously published works, please review and evaluate these publications to determine whether they are relevant and should be cited. There is no requirement to cite these works unless the editor has indicated otherwise.

We have revised the manuscript according to the editor and the reviewers’ feedback.

- Additional Editor Comments :

1. Severe adverse events were defined as in-hospital death, ICU admission, diagnosis of sepsis or use of extracorporeal membrane oxygenation. Please specify the criteria used to define these, and what about the need for vent support, RRT, etc.

When we designed this study, we conducted a literature search on prognostic studies of biomarkers in both adult and pediatric patients with pneumonia. We found some variations in defining severe short-term outcomes for pneumonia patients. However, most papers used combinations of death, ICU care, sepsis, septic shock, ECMO, mechanical ventilation, vasoactive infusions, chest drainage, respiratory failure, empyema, and ARDS (see, for example, References 4,8,13 and 21 in the manuscript) as outcome factors. For our study, we chose severe adverse events as a composite endpoint, including in-hospital death, ICU admission, diagnosis of sepsis, and use of ECMO—all of which represent death or life-threatening conditions.

1. In-hospital death is death that occurred during hospitalization.
2. In our hospital, a patient is typically admitted to the ICU if at least one of the following conditions is present: (1) FiO2 ≥ 0.6, SaO2 ≤ 0.92, (2) shock and/or impaired consciousness, (3) tachypnea and tachycardia with severe respiratory distress or signs of exhaustions, with or without elevated PaCO2, (4) recurrent apnea, or slow and irregular breathing, and (5) other conditions requiring further monitoring and treatment (e.g. renal failure, severe thrombocytopenia)
3. The definition of sepsis follows the Third International Consensus Definitions for sepsis as life-threatening organ dysfunction caused by a dysregulated host response to infection, where organ dysfunction can be identified as ≥ 2 points on the Phoenix Sepsis Score in children with suspected infection [1].
4. The use of ECMO is mainly indicated for patients requiring respiratory support due to: (1) PaO2/FiO2 < 60-80 mmHg due to severe respiratory failure; (2) failure of conventional ventilation and/or other rescue therapies; (3) high ventilator settings (e.g., mean airway pressure > 20 - 25 cm H₂O during conventional ventilation or > 30 cm H₂O during high-frequency ventilation, or signs of iatrogenic barotrauma). ECMO is also indicated for circulatory support in cases such as cardiogenic shock unresponsive to standard medication, with low SBP < 50 mmHg, urine volume < 1 ml / (kg·h), lactic acidosis, central venous oxygen saturation < 0.6, altered mental status due to low cardiac output, and refractory septic shock with an epinephrine dose > 1 μg / (kg·min) or vasoactive-inotropic score >100.

Regarding renal replacement therapy and ventilation support, we collected information from medical records. Three patients received renal replacement therapy, and these patients were all admitted to the ICU. 6 patients received ventilation support including ECMO (n=4) and invasive mechanical ventilation (n=2). All of these patients were admitted to the ICU. Therefore, all patients who received mechanical ventilation or renal replacement therapy were included in the severe adverse events group.

We reviewed all the patient data to confirm sepsis diagnosis. Based on the diagnostic criteria, nine patients met the definition for sepsis, and all were included in the severe adverse events group.

The manuscript has been revised accordingly. The definitions have been provided in the supporting document ‘S1 File’ cited in the manuscript.

[1] Schlapbach LJ, Watson RS, Sorce LR, Argent AC, Menon K, Hall MW, et al. International Consensus Criteria for Pediatric Sepsis and Septic Shock. JAMA. 2024;331(8):665-674. doi:10.1001/jama.2024.0179

2. In some patients, sepsis might be mild without MODS, others severe sepsis with MODS. Was there any subgroup analysis performed and why if not?

There are nine sepsis patients in our cohort, among whom eight had multiorgan dysfunction. The most common organ dysfunctions happened in respiration system (n = 9) and coagulation system (n = 8), followed by circulation system (n = 4) and renal system (n = 3). One patient had only respiratory failure and received invasive mechanical ventilation. Due to a small sample size of sepsis patients, we did not perform subgroup analysis.

3. You have mentioned that sST2 levels were significantly higher in severe MPP cases and those with co-infections. If co-infections were detected, what is the specificity for its association with MPP? Sst2 could have been elevated due to other infections as well. How do you explain the uncertainty?

In this study, we have demonstrated that sST2 levels were significantly higher in severe MPP, MPP with complications, and MPP with severe adverse events. We consider sST2 to be a prognostic marker, rather than a diagnostic marker. Due to the biological functions of sST2, high sST2 levels may reflect excessive immune response and severe inflammation.

sST2 is not specific to MPP. Previous studies (Reference 12,27) have shown that sST2 levels increase in pneumonia caused by other pathogens. We found that sST2 levels were higher in MPP with co-infections. A higher proportion of patients with co-infections were in the severe MPP group compared to the proportion of patients with MP infection alone, although this difference did not reach statistical significance (37.3% vs. 23.4%, P = 0.0723). We think this may partly explain the association of sST2 and co-infection.

Meanwhile, among the patients with only MP infection, sST2 levels in severe MPP patients were higher than non-severe MPP (30.8 [14.5, 75.4] vs 22.6 [12.8, 52.0] ng/mL, P=0.361), although it did not reach statistical significance, possibly due to a relatively small sample size. Further, among the patients with only MP infection, three patients had severe adverse events and their sST2 levels (49.2, 145.2, and 189.7 ng/mL respectively) were significantly higher than the rest (22.6 [12.8, 52.0] ng/mL), but the sample size is very small. These findings warrant further validation in a larger cohort.

We will conduct a prospective study to evaluate the role of sST2 in children of community acquired pneumonia caused by different pathogens including MP with a larger sample size in the future.

4. You have mentioned that sST2 levels correlated positively with neutrophil count, neutrophil to lymphocyte ratio, PCT, CRP and IL-6- comparative analysis. Was sST2 more specific, more sensitive, cost effective, etc. What is the distinct advantage of doing Sst2, compared to performing NLR, ANC, PCT, CRP?

In this study, we aimed to demonstrate the value of sST2 in predicting severe adverse events as a prognostic marker. Through comparisons of ROC AUCs, we found that sST2 had better performance in predicting severe adverse events in this cohort. Its AUC was significantly higher than those of CRP and NLR, and higher than those of PCT and IL-6 without statistical significance. Furthermore, in our cohort, sST2 was the only independent biomarker associated with severe adverse events in a multiple logistic regression analysis which included sST2, PCT and IL-6 as independent variables (see Results—Associations of sST2 with severe adverse events during hospital stay). Therefore, we think sST2 may predict severe adverse events more effectively than conventional inflammatory biomarkers. However, this retrospective study has several limitations as we have mentioned in our manuscript, therefore we plan to confirm these findings in a prospective study with a larger sample size.

5. Lines 49-50: Studies reported mixing findings on accuracy of this method to predict patients’ outcome and inter-physician variations [4,5]- Grammar error needs to be corrected.

We corrected it as “Previous studies have reported mixed results regarding the accuracy of this approach as well as substantial inter-physician variability”.

6. What is authenticity of definition of severe MPP? Your aim was to evaluate a biomarker whether it predicts severity. If only 4 parameters are assessed, there is a large gap of knowledge about those patients who were oxygen dependent, MODS but did not require organ support, those left with complications, destroyed lungs, etc.

The diagnosis for MPP and severe MPP were established in accordance with the Chinese guideline for the diagnosis and treatment of childhood Mycoplasma pneumoniae pneumonia (2023) (Reference 1, 2). This guideline was issued by the National Health Commission of the People's Republic of China.

The definition of severe MPP is provided in the ‘Materials and Method- Clinical data collection section’, as ‘MPP patients with any of the following criteria[1,2]: (1) high fever (> 39°C) for more than 5 days or fever for more than 7 days without a declining trend in peak temperature; (2) hypoxemia (maintained an SaO2 < 92% on room air); (3) increasing respiratory and pulse rates with clinical evidence of respiratory distress and exhaustion with or without a elevated PaCO2; (4) signs of intrapulmonary infection, such as moderate to large pleural effusion, large area of pulmonary consolidation, plastic bronchitis, pulmonary embolism, necrotizing pneumonia, and acute asthma exacerbations; and (5) signs of extrapulmonary complications, such as meningoencephalitis, ascending (i.e., Guillain-Barré) paralysis, myopericarditis, erythema multiforme, autoimmune hemolytic anemia, hemophagocytic syndrome, or disseminated intravascular coagulation’ According to the above definition, the MPP patients with complications, and destroyed lungs requiring oxygenations are included in severe MPP.

Although our primary aim is to evaluate the association of sST2 with severe adverse events, we evaluated its association with clinical characteristics, including severe MPP. We found that sST2 levels in the severe MPP patients were significantly higher (71.91 [30.76-140.12] vs 26.2 [15.15-54.69], P < 0.001). Among the severe MPP patients, sST2 levels were significantly higher in those with severe adverse events compared to those without (170.7 [132.5, 246.3] vs. 45.1 [18.0, 90.7] ng/mL, P < 0.001).

7. Lines 83-84: The diagnosis of SMPP is made in MPP patients with either of the following criteria [2]- Grammar error needs to be corrected.

We corrected it as “Severe MPP was defined as MPP with any of the following criteria”.

8. Line 140: media hospital stay was 7 days- grammar error

We corrected it as “median hospital stay”.

9. Line 142: ECOM- spelling error needs to be corrected.

We corrected it as “ECMO”.

10. The study design is ambiguous- criteria for severe MPP is delineated, however, the parameter Sst2 was evaluated only for 12 patients who had fit into 4 separate set of “adverse events”. These adverse events are overlapping over the defined criteria. It is unclear about sst2 levels among the actual population of severe MPP patients who qualified based on set criteria [2]. The study is actually assessing predictive capacity of sst2 to identify severe adverse events among MPP rather than severity of MPP.

The primary aim of this study is to evaluate the association of sST2 with severe adverse events, which were selected based on a literature search as discussed above. While some patients experienced multiple adverse events, this was not always the case.

Severe MPP patients had significantly higher sST2 levels compared to non-severe patients (71.91 [30.76–140.12] vs. 26.2 [15.15–54.69] ng/mL, P < 0.001) (see Results—Correlation between sST2 and other laboratory biomarkers and clinical parameters).

We agree that the study primarily aims to assess the predictive capacity of sST2 for identifying severe adverse events in MPP—rather than MPP severity alone. However, we also demonstrated that sST2 was associated with severe MPP as defined by established criteria, which may partly explain the association of sST2 and severe adverse events. We have revised the abstract to make it clearer.

11. Line 229: Consequently, MP infection undergone a prolonged non-seasonal epidemic, which emerged in April 2023- grammar error needs correction.

We corrected it as “Consequently, MP caused a prolonged non-seasonal epidemic”.

12. Title: Soluble ST2 is a potential biomarker for risk stratification of pediatric patients with mycoplasma pneumoniae pneumonia- better stated as sst2 as a biomarker for predicting severe adverse events among pediatric patients with MPP.

We agree to change the title to “Soluble ST2 as a biomarker for predicting severe adverse events among pediatric patients with Mycoplasma pneumoniae pneumonia”.

- Reviewer’s comments
  - Reviewer #1: The study explores the association of sST2 with in-hospital adverse events and clinical characteristics in pediatric MPP patients, and assesses its potential as a prognostic biomarker in comparison with conventional inflammatory markers. The work is sound but requires major revisions before publication.

Methods

1. A brief description of the study setting will be appropriate. Clarify if it's routine to have sST2 as a baseline investigation for MPP in the hospital.

The sST2 assay was a newly introduced test during the study period. It was prescribed at the attending physician's discretion.

2. Sampling: Throw more light on sampling. How many confirmed MPP in the study period, and how many were excluded. Include a flow diagram (as per PLOS ONE guidelines) showing total confirmed cases of MPP recorded in the study period, how many folders were reviewed, how many excluded, and the final sample analyzed.

The sST2 assay was a newly introduced test during the study period and was prescribed at the attending physician's discretion. During this period, 3,794 patients with MPP were hospitalized, of whom 205 had sST2 results. Of these, 58 patients were excluded due to the following reasons: age or hospital stay length (n = 4), sST2 assay performed at a late time point (n = 19), congenital heart diseases (n = 6), autoimmune diseases (n = 4), cancer (n = 3), and active bronchial asthma or pulmonary tuberculosis (n = 22). Finally, 147 patients were included in this retrospective study. The medical records of all these patients were complete. We reviewed the medical records of all these 147 patients and extracted data. The sampling process has been added to Materials and methos-subjects section. The flow diagram is provided as S1 Fig.

3. Clearly define and specify (primary versus secondary outcome variables, factor variables) all variables. Consider listing all variables, measurement methods, and units for clarity.

We included a ‘Study variables’ subsection in the Materials and Methods section. The outcome variable of this study was severe adverse events, defined as in-hospital death, ICU admission, diagnosis of sepsis or use of ECMO (S1 File). The primary predictor variable was sST2, and the covariates included demographic variables (age, sex) as well as other blood biomarkers (CRP, PCT, IL-6, WBC, LYM, NEU and NLR).

The units for all biomarkers are provided in Table 1. Their measurement methods are described in Materials and methods - Laboratory tests section.

4. Clarify how missing data were handled.

For all 147 patients included in this study, their clinical and laboratory data are complete. All the other blood biomarkers are routinely measured for pneumonia patients hospitalized in our hospital, and there are no missing data. Accordingly, we added the following statement to the Materials and methods - Statistical analysis section: " All analyses were performed on complete observations given that there were no missing values present in the data."

Analysis and Results

5. The whole table 1 lacks clarity. Some entries do not correspond with the labelling of the columns or rows. Authors should clarify why Table 1 contains ‘severe adverse event’ in both rows and columns. Also, the decimals with excess zeroes are not necessary for frequencies in the table. Clearly specify which values represent mean (sd), median (iqr), or frequency (%), in the table. Define what constitutes ‘other infections’ in the table?

We have removed the rows for severe adverse events and the decimals for categorical variables. As described in the Materials and Methods—Statistical analysis subsection and the footnote of Table 1, all continuous variables are presented as median (interquartile range) and categorical variables as numbers (%). Regarding other infections, they were fungal infections. Therefore, we changed 'other infections' to 'fungal infection'.

6. Format and standardize the table and ensure statistical reporting follows PLOS ONE’s reporting standards.

We have reformatted the tables and checked the statistical reporting according to the PLOS One submission guidelines.

7. The question remains, what is/are your primary outcome measure (s) (end-point (s))? Severe MPP? Severe adverse events? Complications?

The outcome variable is severe adverse events, including in-hospital death, ICU admission, diagnosis of sepsis, or use of ECMO. We have added this information to the Materials and Methods – study variables section.

8. Consider fitting logistic regression for Severe MPP if it is considered a primary outcome.

The outcome variable of this study is severe adverse events. We evaluated the association of sST2 with severe MPP, which may partly explain the association of sST2 and severe adverse events.

9. Recommendation: Your introduction section should include a conceptual framework to clarify the multiple relationships under study. For instance, how presenting disease severity may be associated with high levels of sST2, how sST2 may in turn be related to in-hospital adverse events, and/or complications.

We have revised the introduction section as suggested by the reviewer.

Discussion

10. The discussion requires focus and direction. Provide clearer comparisons with existing evidence, focusing more on the association between sST2 and the primary outcome(s).

We have revised the discussion section as suggested by the reviewer.

Minor

11. Minor typographical and grammatical errors include wrong spellings and tenses (E.g. Line 62: Studies have shown and not ‘have showed’), missing letters; the whole manuscript requires proofreading before resubmission.

We have corrected the typographical and grammatical errors.

- Reviewer #2: This study evaluates soluble ST2 (sST2) as a prognostic biomarker in pediatric Mycoplasma pneumoniae pneumonia and addresses a clinically important and timely question. The authors show that sST2 is strongly associated with disease severity, complications, and in-hospital adverse events, with superior predictive performance compared to several conventional inflammatory markers. The study is well conducted and biologically plausible; however, its retrospective single-center design, small number of severe adverse events, and lack of clinical severity indices limit generalizability and warrant cautious interpretation. Overall, the findings are promising and support the need for larger, prospective, multicenter studies to validate sST2 for clinical risk stratification.

Thank you for the comments. We agree that this single-center retrospective study has several limitations which limit the generalizability of the findings. In the future, we plan to conduct a multi-center prospective study with a larger study population and more comprehensive clinical data to further validate the clinical value of sST2 in children with MPP and other pneumonia. We have revised the study limitations in the Discussion section.

- Reviewer #3: Introduction

The Introduction is relevant but needs improvement

1. Makes Sentences more clear and simple ,

We have revised the introduction section as suggested by the reviewer.

2. Avoid using terms like “clinical gestalt” and better to be replaced with standard language,

We have revised the sentence to make it clearer: “Currently, physicians often rely on clinical judgement based on overall patient assessment to distinguish high-risk patients.”

3. Prefer to use more neutral tone and avoid assertive, strong statements

We have revised the introduction section with more neutral tone, as suggested by the reviewer.

4. The research gap is clearly defined; but could be strengthened by briefly stating what is unknown about sST2 in pediatric MPP.

We have revised the introduction section by adding more details of the research gap as suggested by the reviewer.

5. Correction of minor language and formatting issues also needed

We have corrected the typographical and grammatical errors. The manuscript has been reformatted according to the PLOS One submission guidelines.

Materials and Methods

The Materials and Methods section is looks clear,

6. clarification of the study timeline should be done.

The retrospective analysis was conducted between June 2024 to January 2025 on the patients who were hospitalized between April 2023 and May 2024. This has been provided in ‘Materials and methods- subjects’ section.

7. explain the reasons for exclusion criteria,

The exclusion criteria were patients with (1) congenital heart diseases, (2) autoimmune diseases, (3) cancer, (4) active bronchial asthma or pulmonary tuberculosis, and (5) incomplete medical records.

Our main focus in this study was the clinical value of sST2 in MPP, particularly as an inflammatory biomarker. The rationale for each exclusion criterion is as follows:

- Congenital heart diseases: Since sST2 is known to be involved in cardiac diseases, we excluded patients with congenital heart diseases, which are the most prevalent heart conditions in children.
- Autoimmune diseases and asthma: sST2 is an inflammatory biomarker involved in immune regulation, and studies have reported altered sST2 levels in autoimmune diseases such as systemic lupus erythematosus (SLE), inflammatory bowel disease (IBD), and rheumatoid arthritis (RA), as well as in asthma.
- Cancer: Given that cancer has a major impact on prognosis, patients with malignancy were excluded.
- Pulmonary tuberculosis: Pulmonary tuberculosis is an infectious disease distinct from community-acquired pneumonia in terms of disease course, pathophysiology, treatment, and prognosis; therefore, these patients were excluded.

8. specify the source of diagnostic guidelines

The diagnosis criteria of MPP and severe MPP are based on the Chinese guideline for the diagnosis and treatment of childhood mycoplasma pneumoniae pneumonia (2023) by the National Health Commission of the People's Republic of China. (Reference 1,2)

9. details on the laboratory methods used should be provided especially information about sST2 measurement

The measurement methods for all blood biomarkers have been provided in the Materials and Methods—Laboratory tests section. All clinical assays and analyzers used in this study are approved by the China National Medical Products Administration (NMPA).

10. make the ethics statement more concise and properly formatted.

We have added an ‘Ethics statement’ subsection.

The Clinical Data Collection

provide basic descriptions but lack essential methodological details.

11. provide more details about The data collection and extraction, how complications and adverse events were defined and classified.

A total of 147 patients were included in this retrospective study based on the predefined inclusion and exclusion criteria. We then reviewed each patient's medical records to extract data on demographic characteristics, pathogens, severe MPP, physician-diagnosed conditions, ICU admissions, and in-hospital deaths. Severe adverse events and pulmonary and extrapulmonary complications were then classified according to predefined criteria.

Severe adverse events include in-hospital death, ICU admission, diagnosis of sepsis and use of extracorporeal membrane oxygenation. The definitions are as follows:

1) In-hospital death is death that occurred during hospitalization.
2) In our hospital, a patient is typically admitted to the ICU if at least one of the following conditions is present: (1) FiO2 ≥ 0.6, SaO2 ≤ 0.92, (2) shock and/or impaired consciousness, (3) tachypnea and tachycardia with severe respiratory distress or signs of exhaustions, with or without elevated PaCO2, (4) recurrent apnea, or slow and irregular breathing, and (5) other conditions requiring further monitoring and treatment (e.g. renal failure, severe thrombocytopenia)
3) The definition of sepsis follows the Third International Consensus Definitions for sepsis as life-threatening organ dysfunction caused by a dysregulated host response to infection, where organ dysfunction can be identified as ≥ 2 points on the Phoenix Sepsis Score in children with suspected infection [1].
4) The use of ECMO is mainly indicated for patients requiring respiratory support due to: (1) PaO2/FiO2 < 60-80 mmHg due to severe respiratory failure; (2) failure of conventional ventilation and/or other rescue therapies; (3) high ventilator settings (e.g., mean airway pressure > 20 - 25 cm H₂O during conventional ventilation or > 30 cm H₂O during high-frequency ventilation, or signs of iatrogenic barotrauma). ECMO is also indicated for circulatory support in cases such as cardiogenic shock unresponsive to standard medication, with low SBP < 50 mmHg, urine volume < 1 ml / (kg·h), lactic acidosis, central venous oxygen saturation < 0.6, altered mental status due to low cardiac output, and refractory septic shock with an epinephrine dose > 1 μg / (kg·min) or vasoactive-inotropic score >100.

These definitions are provided in S1 File.

Pulmonary and extrapulmonary complications were defined according to the Chinese guidelines for the management of community-acquired pneumonia in children (2024 revision) [2].

Pulmonary complications included acute respiratory distress syndrome (ARDS), respiratory failure, pulmonary necrosis, pleural effusion, plastic bronchitis, pulmonary embolism, and pulmonary atelectasis.:

1) Acute respiratory distress syndrome is diagnosed according to the PALICC-2 criteria [3]. Respiratory failure must develop within 7 days of a known clinical insult, which cannot be fully explained by cardiac failure or fluid overload. The presence of new parenchymal opacities on chest imaging is not due to atelectasis or effusion. Hypoxemia is stratified by the mode of respiratory support. For patients on invasive mechanical ventilation, hypoxemia is assessed using the Oxygenation Index ≥ 4 or Oxygen Saturation Index ≥ 5. For those on non-invasive ventilation (with a full-face interface and CPAP PEEP ≥ 5 cm H₂O) or high-flow nasal cannula, hypoxemia is diagnosed based on PaO₂/FiO₂ ≤ 300 or SpO₂/FiO₂ ≤ 250.
2) Respiratory failure is diagnosed: (1) underlying etiology—identification of the primary or contributory condition causing respiratory failure; (2) clinical manifestations—presence of increased work of breathing (e.g., tachypnea with or without cyanosis) or irregular breathing patterns; (3) arterial blood gas (ABG) criteria — for Type I (hypoxemic) respiratory failure, PaO₂ < 60 mmHg with normal or low PaCO₂; for Type II (hypercapnic) respiratory failure, PaO₂ < 60 mmHg and PaCO₂ > 50 mmHg. For patients on supplemental oxygen, a PaO₂/FiO₂ ratio < 200 mmHg suggests significant intrapulmonary shunting (>20%), while a ratio > 300 mmHg is generally considered normal.
3) Pulmonary necrosis is diagnosed via CT examination, showing multiple thin-walled cavities with low density, containing air or fluid, occurring in areas of pulmonary consolidation.
4) Pleural effusion is diagnosed via chest ultrasound, CT or X-ray examination. Imaging findings include blunting of the costophrenic angle on X-ray, anechoic or septated fluid collections on ultrasound, and crescent-shaped opacities in the dependent pleural space on CT.
5) Plastic bronchitis is diagnosed based on the expectoration or bronchoscopic removal of bronchial tree-shaped casts.
6) Pulmonary embolism is diagnosed based on CT pulmonary angiography showing filling defects within the pulmonary arteries, reduction or absence of distal vascular branches, and wedge-shaped pulmonary lesions.
7) Pulmonary atelectasis: Diagnosed via CT scan, showing signs of volume loss, such as displacement of interlobar fissures toward the area of collapse, crowding of pulmonary vessels and bronchi within the affected region, and compensatory hyperinflation of the remaining lung tissue.

Extrapulmonary complications were classified as follows:

1) Nervous system involvement includes encephalitis, acute disseminated encephalomyelitis, transverse myelitis, Guillain-Barré syndrome, and cerebral infarction, with encephalitis being the most common. These conditions should be considered when children with pneumonia present with symptoms such as convulsions or altered consciousness.
2) Circulatory system involvement includes intracardiac thrombosis, septic shock, myocarditis, pericarditis, Kawasaki disease, arterial embolism, and venous thrombosis. These conditions should be considered when children with pneumonia have abnormal cardiac biomarkers. Particular attention should be paid to these complications in children with severe or refractory MPP.
3) Hematological system involvement includes immune thrombocytopenia, autoimmune hemolytic anemia, hemophagocytic syndrome, and disseminated intravascular coagulation. These complications should be considered when there is a significant decrease in peripheral blood cell lines.
4) Skin and mucosal lesions include urticaria, Henoch-Schönlein purpura, erythema multiforme, Stevens-Johnson syndrome, toxic epidermal necrolysis, and Mycoplasma pneumoniae-induced rash and mucositis (MIRM).
5) Other manifestations include glomerulonephritis, acute kidney injury, liver failure, acute pancreatitis, arthritis, and rhabdomyolysis.

We have provided the definitions and classifications of complications and severe adverse events in supporting document ‘S1 file’ cited in the manuscript.

[1] Schlapbach LJ, Watson RS, Sorce LR, Argent AC, Menon K, Hall MW, et al. International Consensus Criteria for Pediatric Sepsis and Septic Shock. JAMA. 2024;331(8):665-674. doi:10.1001/jama.2024.0179

[2] Subspecialty Group of Respiratory, the Society of Pediatrics, Chinese Medical Association; Editorial Board, Chinese Journal of Pediatrics; China Medicine Education Association Committee on Pediatrics. [Guidelines for the management of community-acquired pneumonia in children (2024 revision)]. Zhonghua Er Ke Za Zhi. 2024;62(10):920-930. doi:10.3760/cma.j.cn112140-20240728-00523

[3] Emeriaud G, López-Fernández YM, Iyer NP, Bembea MM, Agulnik A, Barbaro RP, et al. Executive Summary of the Second International Guidelines for the Diagnosis and Management of Pediatric Acute Respiratory Distress Syndrome (PALICC-2). Pediatr Crit Care Med. 2023;24(2):143-168. doi:10.1097/PCC.0000000000003147

12. Some outcomes like sepsis needs to be referenced to established criteria,

The definition of sepsis follows the Third International Consensus Definitions for sepsis as life-threatening organ dysfunction caused by a dysregulated host response to infection, where organ dysfunction can be identified as ≥2 points on the Phoenix Sepsis Score in children with suspected infection [1]. We have added the diagnosis criteria for sepsis in the supporting document S1 File cited in the manuscript.

[1] Schlapbach LJ, Watson RS, Sorce LR, Argent AC, Menon K, Hall MW, et al. International Consensus Criteria for Pediatric Sepsis and Septic Shock. JAMA. 2024;331(8):665-674. doi:10.1001/jama.2024.0179

13. More clear categorization of the complications is needed. cardiac complications—such as myocarditis, pericarditis, arrhythmias, and heart failure—should be clearly highlighted, given some evidence linking soluble ST2 to cardiac injury in recent studies.

Cardiac complications were classified as circulatory system complications, along with other conditions such as intracardiac thrombosis, septic shock, Kawasaki disease, arterial embolism, and venous thrombosis. In our study, 10 patients had circulatory system complications, including six with abnormal cardiac biomarkers, two with tachycardia, one with septic shock, and one with myocarditis. sST2 levels of these patients were significantly higher than the rest (107.15 [91.88–178.97] vs. 34.75 [15.74–72.21] ng/mL, P < 0.001).

Laboratory Tests

14. Provide information on sample timing, assay characteristics, units, and data handling.

Laboratory test results including sST2, CRP, PCT, IL-6, WBC, LYM, NEU and NLR at admission were collected from medical records. The units for all biomarkers are provided in Table 1, and their measurement methods are described in Laboratory tests subsection.

15. Both subsections require clearer definitions, standardized terminology

We have revised these sections to improve clarity.

16. Correct grammatical and formatting issues

We have corrected grammatical errors and reformatted the manuscript according to the PLOS one submission guidelines.

Results

The Results section contains valuable data but

17. Sentence needs to be more clear with correction of the language errors

- Write the result with more direct and objective way
- More clear and correct terminology should be used
- Tables and figures need better organization, and more detailed sand clear tatistical reporting is needed

We revised the results section according to the comments of the reviewer.

Discussion

18. Although the discussion is well-organized, but should be written in more clear, direct objective way aligning with the nature of this single-center, retrospective study.

- Present any potential therapeutic or predictive outcomes as theoretical possibilities or areas for future investigation, not as current treatment protocols or guaranteed outcomes.
- Conclusions avoid statement that indicate accuracy and superiority.
- limitations should be extended to properly reflect design and analytical difficulties.

We revised the discussion section according to the comments of the reviewer.

- To ensure your figures meet our technical requirements, please review our figure guidelines: https://journals.plos.org/plosone/s/figures

You may also use PLOS’s free figure tool, NAAS, to help you prepare publication quality figures: https://journals.plos.org/plosone/s/figures#loc-tools-for-figure-preparation.

NAAS will assess whether your figures meet our technical requirements by comparing each figure against our figure specifications.

We have reformatted the manuscript according to the PLOS One submission guidelines. The figures were generated using NAAS.

In compliance with data protection regulations, you may request that we remove your personal registration details at any time. (Remove my information/details). Please contact the publication office if you have any questions.
